# Extrusion Techniques—Clinical Applications of Modern Tooth Preservation Concepts and Their Comparison

**DOI:** 10.1111/jerd.13486

**Published:** 2025-05-19

**Authors:** Elisabeth Völler, Michael Stimmelmayr, Jan‐Frederik Güth, Stefan Neumeyer, Daniel Edelhoff, Pauline Gutmann, Tobias Graf

**Affiliations:** ^1^ Department of Prosthetic Dentistry Center for Dentistry and Oral Health, Goethe‐University Frankfurt Frankfurt am Main Germany; ^2^ Department of Prosthetic Dentistry University Hospital, LMU Munich Munich Germany; ^3^ Private Practice Eschlkam Germany

**Keywords:** forced orthodontic extrusion, forced surgical extrusion, magnetic extrusion, orthodontic extrusion ferrule design, restorative dentistry, surgical extrusion, tooth preservation

## Abstract

**Objectives:**

To present and compare several extrusion techniques in case of insufficient tooth structure to create a sufficient ferrule and consider the biological width.

**Clinical Considerations:**

Extrusion methods based on orthodontic or surgical techniques are commonly applied, well‐described, and predictable extrusion methods. Elastics with the highest possible tensile forces, magnets, or orthodontic appliances (e.g., braces) can be used for orthodontic approaches. These procedures are minimally invasive and the extrusion distance to be achieved depends on the forces applied. For the surgical extrusion method, a vertical extraction system is used, and respective teeth are placed in a more coronal position within one appointment. The correct selection of the extrusion method depends on various factors, including inter alia the condition of the periodontal tissues and of the adjacent teeth, as well as the experience and preference of the clinicians. Nevertheless, the presented treatment options require a high level of patients compliance.

**Conclusions:**

Various extrusion techniques enable saving and restoring teeth with severely damaged crowns. They appear to deliver satisfying treatment results with low complication rates, even in the esthetic area. Medium‐term follow‐up data up to 5 years of the concepts are partially available and promising; results on long‐term data are still pending.

## 1 Introduction

The primary focus of nearly all dental interventions should be the long‐term preservation of natural teeth. However, teeth with a high level of coronary destruction, for example, due to secondary caries, traumata, fractures, or resorption processes, regularly present a challenge for clinicians in their daily routine. For such teeth with highly questionable prognosis due to insufficient coronary tooth structure to anchor restorations, clinicians often decide to remove those severely destroyed teeth, followed by fixed dental prostheses (FDPs) or implant‐supported restorations. Despite this, minimally invasive treatment options are also described. The decision between extraction and tooth preservation should be based on an overall treatment plan considering various clinical factors such as the strategic position, endodontic status, surrounding hard‐ and soft‐tissues, condition of the adjacent teeth, remaining tooth structure, fracture line, or patient compliance [1, 2, 3].

In case of delivering compromised teeth with a prosthetic restoration, a circumferential remaining tooth wall height of at least 2 mm is recommended for the ferrule design to ensure a sufficient long‐term fracture resistance [4]. So, for achieving a complete ferrule effect, subgingival preparation margins can be necessary for those destructed teeth. The “dentogingival complex,” describes the combined width of connective tissue and junctional epithelial attachment formed adjacent to a tooth and superior to the crestal bone. Thus, this vestibular distance of 3 mm usually allows for 1 mm of supracrestal connective tissue attachment, 1 mm of junctional epithelium, and 1 mm for sulcus depth [5]. The “biological width” (distance between restorative margin and crestal bone level) needs to be considered during tooth restoration and limits the possibility of creating sufficient ferrule without causing a retraction of crestal bone levels.

Infringements of the supracrestal connective tissue attachment by restorative margins can be associated with inflammatory processes, gingivitis, alveolar bone resorption, loss of periodontal supporting tissue, and poor esthetics [5, 6, 7, 8]. If the distance between the preparation margin and the alveolar bone is less than about 3 mm, there is a high risk of damaging the supracrestal connective tissue attachment [5]. The combined consideration of the ferrule effect and the biological width often confronts the clinician with problems in achieving long‐term complication‐free conditions for severely damaged teeth.

For maintaining the integrity of the supracrestal connective tissue attachment and ensuring the “ferrule effect”, various clinical options are available: surgical crown lengthening as the “surgical invasive” reduction of alveolar bone or less invasive extrusion methods of the remaining root [9, 10]. In the latter case, sufficient tooth wall height and root length should be achieved by moving the tooth stump coronally from the alveolar socket. Therefore, orthodontic or surgical extrusion techniques can be conducted in clinical daily practice. Following, four common methods—these are magnetic extrusion, extrusion with orthodontic appliances, forced orthodontic extrusion (FOE) with maximum forces applied, and forced surgical extrusion—were presented, compared with each other, and discussed.

## 2 Description of Techniques

### 2.1 Prerequisites

For all extrusion techniques, the complete removal of the carious tissue and a sufficient endodontic obturation, endodontic treatment, or retreatment of the compromised tooth to be treated is an obligate prerequisite. So, a preoperative radiograph is mandatory to evaluate root length, apical status, endodontic obturation, and height of the alveolar bone. Only an orthodontic multi‐bracket appliance can be used for the extrusion of vital teeth. Irrespective of the selected extrusion technique, the technique is limited to teeth with a conical root form, mostly anterior or premolar teeth, and a healthy, circumferentially intact periodontium is mandatory for successful outcomes. Furthermore, a high patient compliance and informed consent are requested for all extrusion techniques.

### 2.2 Orthodontic Extrusion Methods

#### 2.2.1 Magnetic Extrusion

Based on a magnetic system with two magnets, the aim of magnetic extrusion is to gain supracrestal tooth structure for a sufficient ferrule design while preserving the soft tissue profile and respecting the supracrestal connective tissue attachment. The clinician can precisely control the forces through specific force–distance diagrams of the magnets used and calculate the force level at any time by measuring the distance between the magnets [11]. Due to planning the precise position of the magnets, the magnetic extrusion of a tooth can be controlled in three dimensions [11].

**FIGURE 1:**
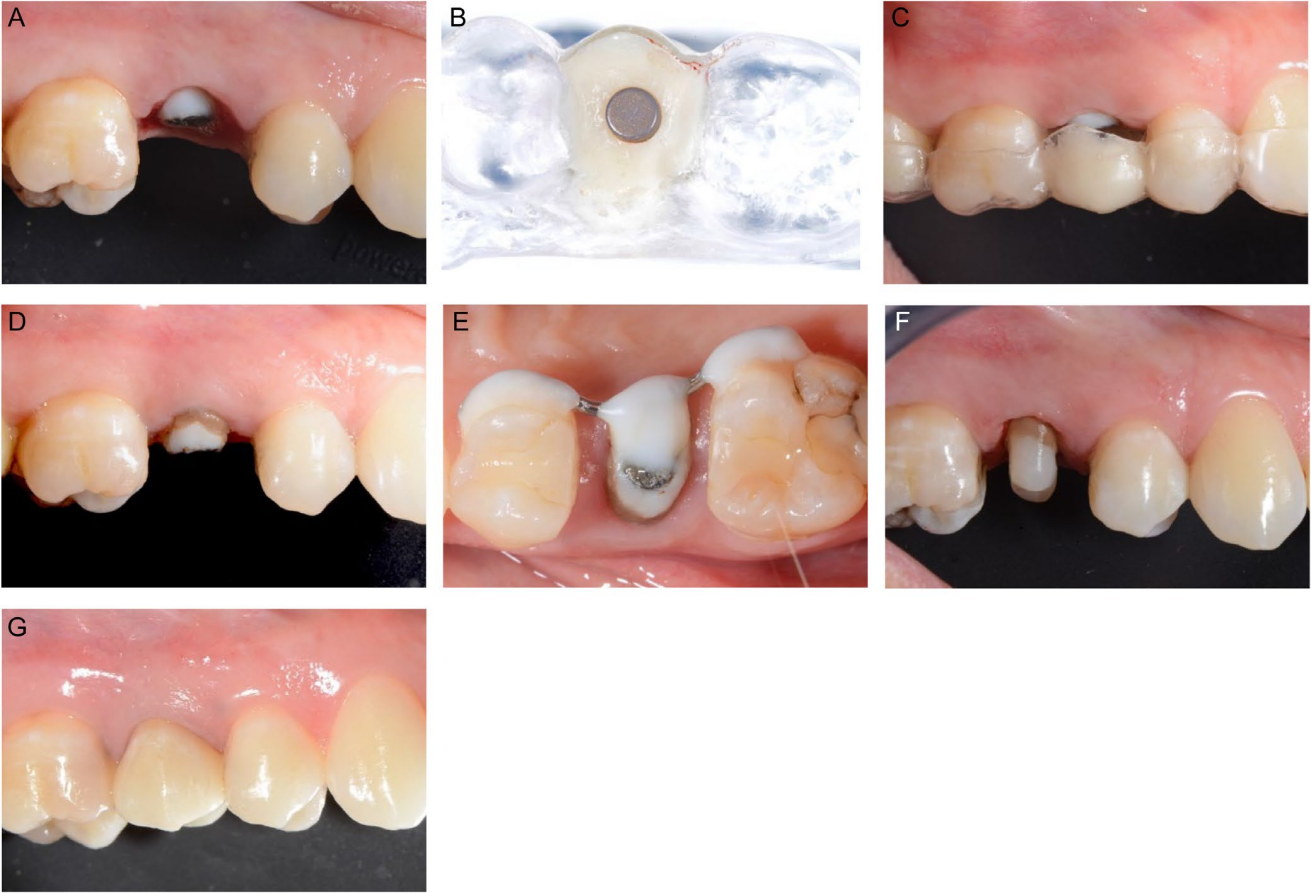
(A–G) Magnetic extrusion.

In the presented case, a disc magnet (e.g., Disc Magnet, Samarium‐Cobalt 5.2 mm × 3 mm) was attached to the root of tooth 25 (FDI notation) parallel to the planned extrusion vector using an adhesive system (Scotchbond Universal Plus, 3M Deutschland GmbH) and a thin‐flowing composite (Figure 1A). The applied forces depend crucially on the distance between the two magnets. An effective working distance of 1 mm between the two corresponding disc magnets can be recommended. For example, an endo stopper might be used as a spacer which is positioned between the magnets. The corresponding magnet disc was pretreated with Monobond Plus (Monobond Plus, Ivoclar Vivadent AG, Schaan) before a vacuum‐formed/thermoformed splint filled with self‐curing composite for provisionals BisGMA (ProTemp, 3M Deutschland GmbH, Neuss) was inserted in the respective region of the magnet in the upper jaw. The spacer between the magnets and excess BisGMA were removed to enable the extrusion process and to facilitate the insertion and removal of the splint. Finally, the solid composite was fixed in the splint using cyanoacrylate (Figure 1B). To prevent migration of the gingiva, the supracrestal sharpey fibers were loosened by fibrotomy. The patient was instructed to wear the splint up to 24 h a day (except for eating). Due to repositioning of the magnet disc in the splint weekly, the root was extruded by 3 mm within 3 weeks in the presented case (Figure 1C).

**FIGURE 2:**
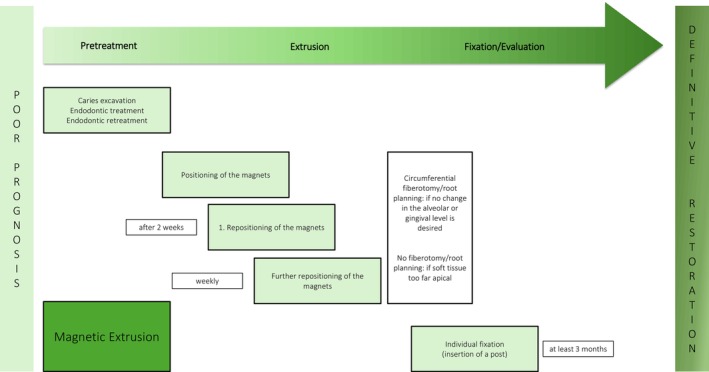
Schematic overview of the magnetic extrusion technique.

If the desired extrusion distance is achieved (Figure 1D), a retention period of 6 weeks was necessary. Here, a customized metal retainer was bonded to the adjacent teeth using composite (Figure 1E). Subsequently, a glass fiber post (d.t. light post, VDW GmbH, Munich, Germany) was inserted and a post‐endodontic build‐up with a bulk fill composite was performed (Figure 1F). A direct provisional (Pro Temp, 3 M Deutschland GmbH, Seefeld, Germany) stayed in situ for another 6 weeks to enable proper healing of soft tissues. After 6 weeks, the definitive crown was inserted (Figure 1G). A schematic overview of this procedure is shown in Figure 2.

There is limited literature on the magnetic extrusion of severely damaged tooth roots. A descriptive retro‐ or prospective study could—to the best knowledge of the authors—not be found in the literature. Some case reports described the method with a follow‐up of up to 3 years. They focused on the magnetic extrusion of tooth stumps for restoration purposes with promising results [8, 11, 12, 13, 14, 15, 16, 17].

#### 2.2.2 Extrusion With Orthodontic Appliances

The conventional orthodontic extrusion works with an orthodontic multi‐bracket appliance, which includes elastics and archwires attached to the teeth, for example as part of orthodontic treatment. This extrusion technique proved to be both effective and stable, with no observable changes to the root or periodontal structures and good functional and esthetic outcomes [18, 19, 20]. In contrast to the other extrusion techniques, a root canal treatment of the tooth to be extruded is not a mandatory prerequisite [21, 22].

**FIGURE 3:**
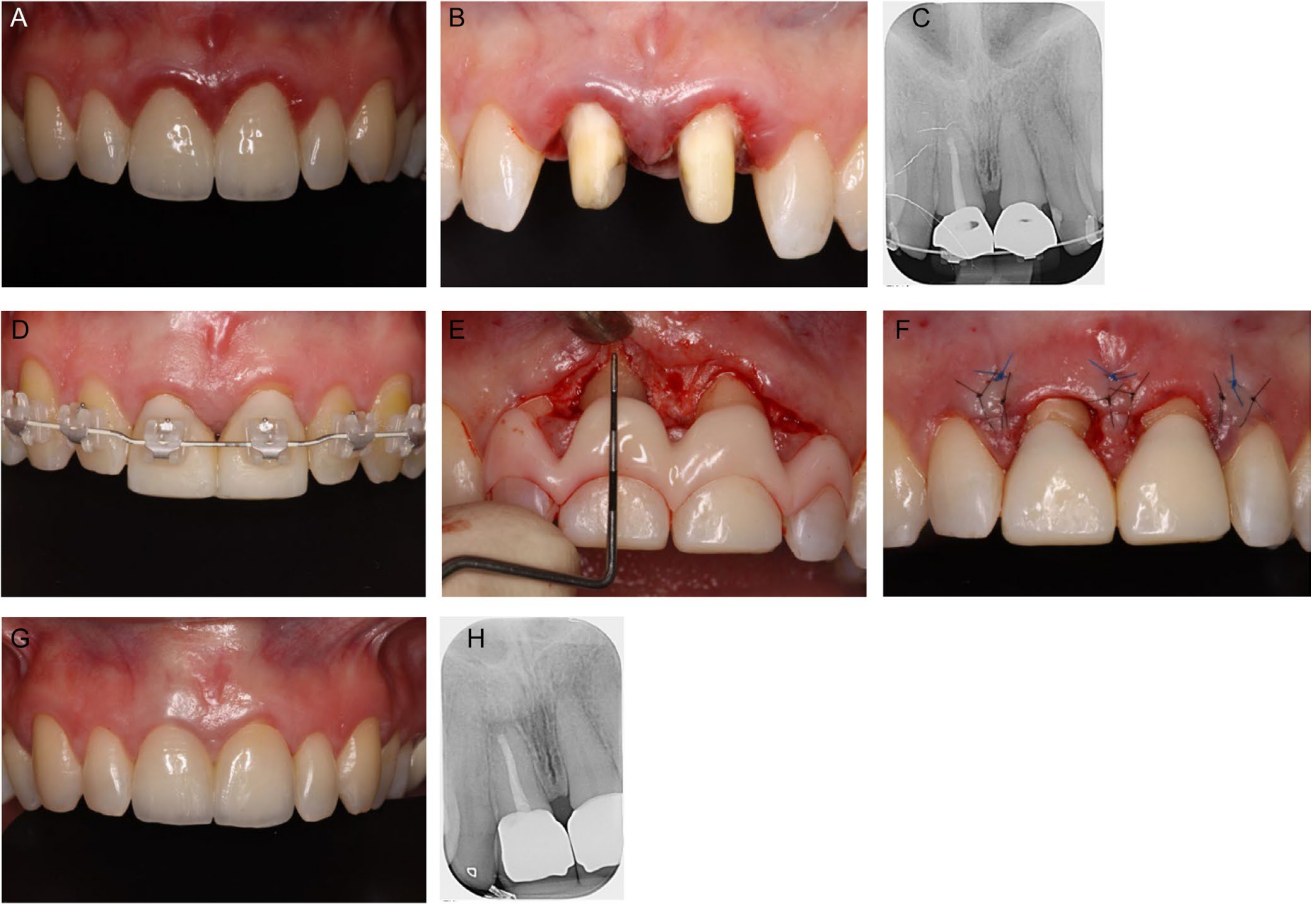
(A–H) Extrusion with orthodontic multi‐band braces.

In the presented case, the teeth 11 and 21 had been restored with complete‐coverage crowns, which had already been replaced twice alio loco. Clinical examination revealed a recurrent gingival inflammation around both teeth (Figure 3A). After crown removal, the subgingival positions of the preparation margins neglecting the biologic width became visible (Figure 3B). First, chairside provisional crowns with supragingival margins were placed for 6 weeks for soft tissue healing. Afterwards, an isogingival preparation was performed and transferred to the dental laboratory by an intraoral scan. Two long‐term provisionals were fabricated and delivered. Orthodontic brackets were passively placed on the buccal surfaces of the adjacent teeth, ensuring no movement of the anchor teeth (Figure 3C,D). For 3 months, a nickel–titanium wire was attached to the brackets [21]. Periodically, the long‐term provisionals were reduced step‐by‐step incisal and palatal to enable the extrusion process. Additionally, a supracrestal fibrotomy was performed to reduce the tension on the sharpey fibers. For a retention period of another 3 months, the wire remained in a passive position before they were removed.

The application of orthodontic extrusion forces resulted in the migration of the root in a coronal direction and in an increased size of the bone ridge and the attached gingiva. So, to harmonize the gingival margin, a surgical crown lengthening was performed. Therefore, the desired preparation margin for the final prosthetic restoration can be evaluated using a resin mockup (Figure 3E).

**FIGURE 4:**
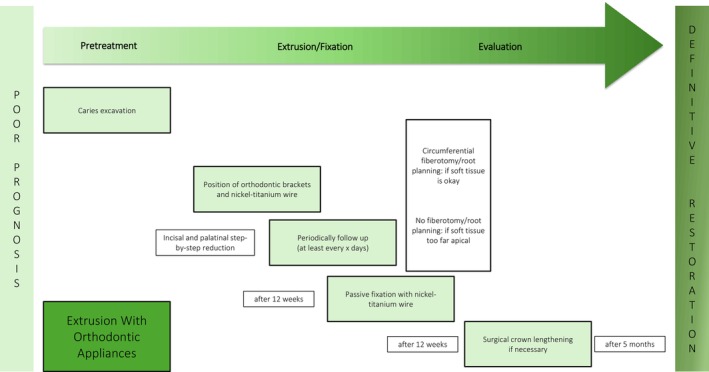
Schematic overview of the extrusion technique with orthodontic appliances.

In the present case the alveolar bone was reduced until a sufficient distance to the preparation margin was achieved while maintaining the biological width (Figure 3F). After a subsequent healing period of 5 months, the final preparation with a circumferential chamfer could be carried out and the prosthetic restoration was inserted (Figure 3G,H). A schematic overview of this procedure is shown in Figure 4.

Extrusion by orthodontic appliances is the preferred choice when a highly predictable treatment is desired, and an orthodontic device is already in use. Furthermore, the method is advantageous if it is important to preserve tooth vitality or manage teeth unsuitable for atraumatic extraction [23]. Rare complications of orthodontic extrusion forces are root resorption, ankylosis, and intrusion of the anchor teeth [24, 25, 26, 27]. Two studies observed no signs of clinical or radiographic inflammation after 7–10 years of follow‐up [28, 29]. However, orthodontic relapse of the extruded tooth is more frequent, which could be prevented by enough retention time and periodically performing supracrestal fibrotomy [30]. However, this could have a disadvantageous effect on the adjacent bone. Carvalho et al. rarely observed osseous resorption after circumferential fibrotomy was done [30].

#### 2.2.3 Forced Orthodontic Extrusion (FOE)

The FOE principle also presents a promising method for preserving teeth as abutments [31]. In contrast to conventional orthodontics, FOE is performed with the highest possible tensile forces, which means that the extrusion distance can be achieved more quickly [32].

**FIGURE 5:**
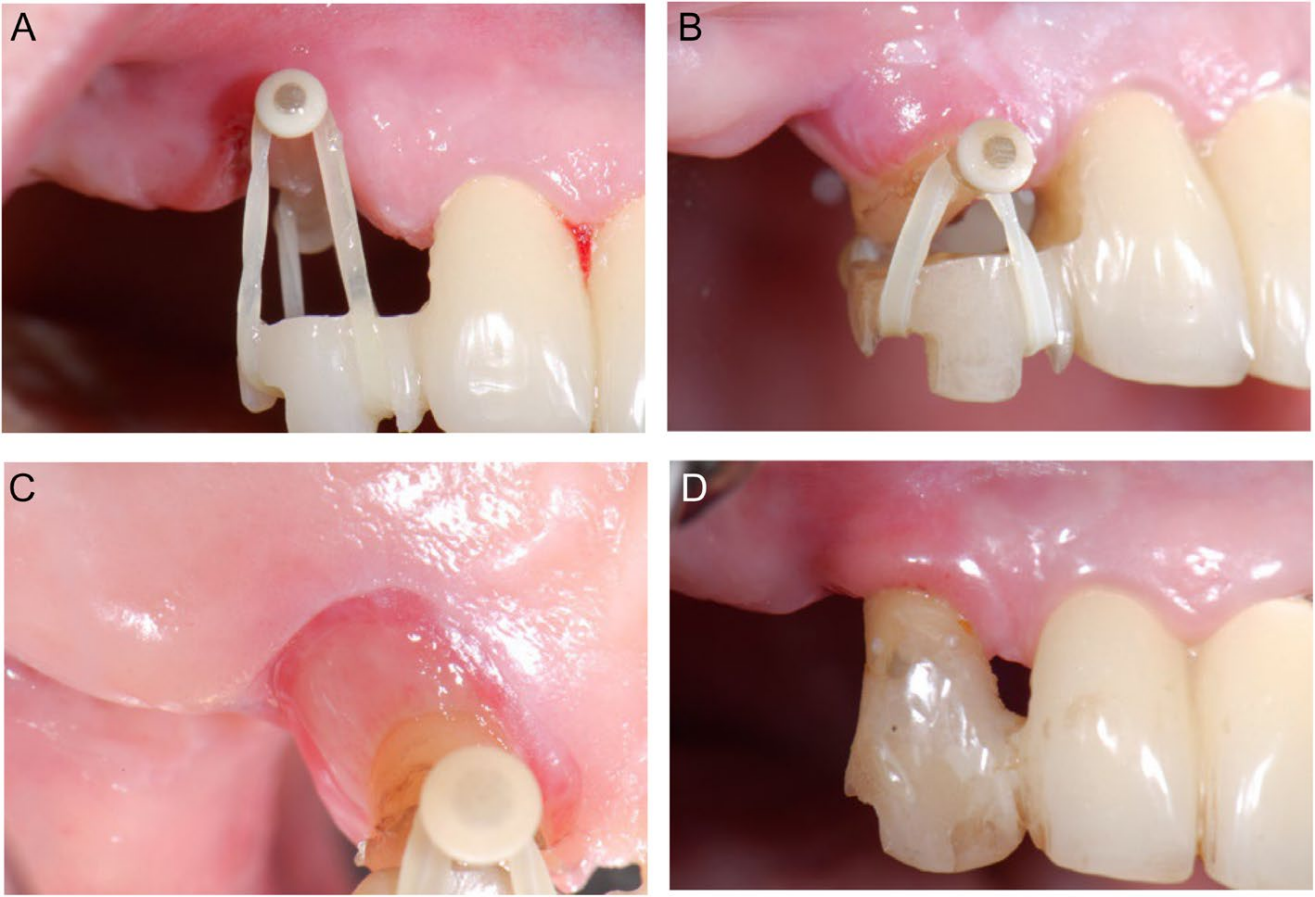
(A–D) Forced orthodontic extrusion.

**FIGURE 6:**
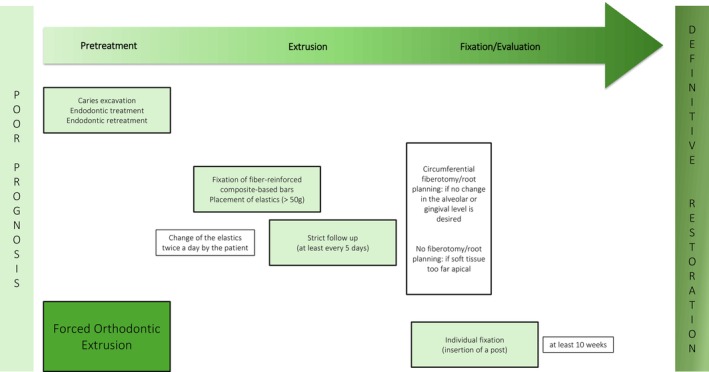
Schematic overview of the forced orthodontic extrusion technique.

In the present case, a fiber‐reinforced composite bar (e.g., Extrusion pin; Gebr. Brasseler GmbH & Co KG, Lemgo, Germany) was adhesively luted to the root surface of the severely compromised tooth 13 using a flow composite. The extrusion anchor was established by a provisional cantilever bridge from 11‐12‐x milled out of PMMA. In the case of healthy adjacent teeth, an additional bar can be used as an anchor, bonded to the neighboring teeth with a flowable composite. The pontic area regio 13 was reduced according to the desired extrusion distance (Figure 5A). Elastics initiate extrusive orthodontic movement with forces of 5–15 N depending on their maximum elasticity. The patient had to replace these elastics twice a day for a rapid extrusion process (Figure 5B). A circumferential supracrestal fibrotomy was not performed at the initial and recall appointments, as the soft tissue should migrate with the extruded tooth (Figure 5C). The amount of extrusion was monitored until the target extrusion distance was achieved, in the present case 6 mm within 11 days. Comparable studies showed that tooth extrusion between 1.3 and 1.8 mm is possible within 7 days, up to extrusion distances of 3.5 (±0.9) mm in 20 (±12) days, without specification of the forces involved [17, 26, 32, 33, 34]. The tooth was splinted to the adjacent teeth for 6 weeks after a chairside adhesive core build‐up (Figure 5D) [17]. After insertion of a glass fiber post (d.t. light post, VDW GmbH) and performing a circumferential ferrule design preparation, the final prosthodontic restoration was placed 3 months after the extrusion. A schematic overview of the FOE procedure is shown in Figure 6.

Clinical evidence on this treatment option also remains scarce and the majority of the existing literature is described in case series and clinical reports over the last two decades [26, 35, 36, 37, 38, 39, 40]. A prospective study with a mean observation period of 3.3 years shows a survival rate of 94% and a success of 84% for teeth extruded by FOE [33]. A clinical study of Bruhnke et al. revealed an overall complication rate of 28%. The most frequent complications of this concept in the extrusive phase were easy to resolve, e.g., loss of adhesion of the composite‐based bar on the root (6%) or adjacent teeth (11%), and consecutive intrusion of the tooth (6%) [31]. In addition, intrusion of the extruded teeth could be observed even at recall appointments later on [33]. Root resorption does not seem to be a relevant side effect of FOE [17, 33]. FOE seems to be a straightforward, time‐saving, and minimally invasive extrusion approach that maintains the surrounding hard and soft tissues [17, 21, 31, 32].

### 2.3 Surgical Forced Extrusion

The fourth technique presented is the surgical forced extrusion using an atraumatic extraction system (AES; e.g., Benex apparatur, Helmut Zepf Medizintechnik, Seitingen‐Oberflacht, Germany) that extrudes the compromised tooth to a coronal required length in one appointment [41, 42, 43, 44].

**FIGURE 7:**
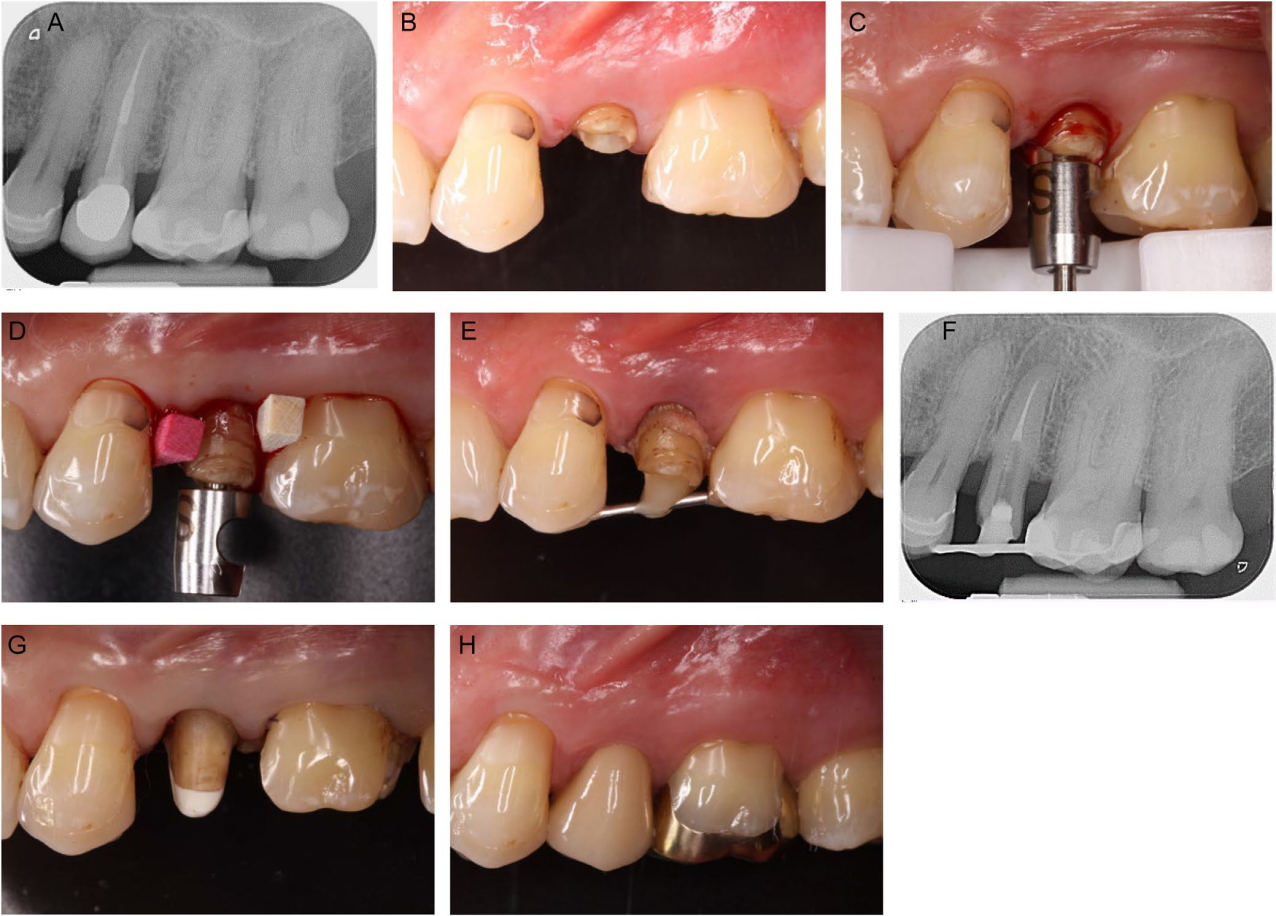
(A–H) Surgical forced extrusion.

**FIGURE 8:**
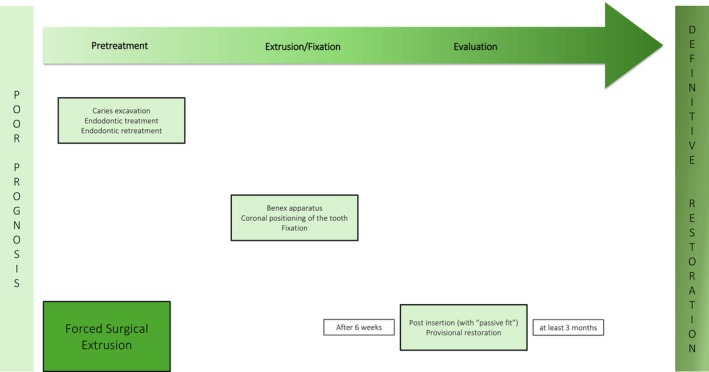
Schematic overview of the surgical forced extrusion technique.

In the present case, the anchor screw of the AES was first screwed in the coronal part of the root canal of tooth 25. Then, the AES device was applied on the adjacent teeth, and the tooth was pulled out to a length for the desired, sufficient ferrule (Figure 7B). In contrast to the approach of other authors [41, 42], the tooth was not fully removed and replanted in a coronal position. The extruded tooth was immediately held in place by two interdental wedges before the anchor screw of the extraction system was removed to avoid any immediate relapse (Figure 7C) and fixed by a wire splint to the occlusally composite filled adjacent teeth in the more coronal position (Figure 7D). After the retention phase of 6 weeks, a root canal post (ER Titanpost, Gebr. Brasseler GmbH & Co. KG), which passively fitted in the prepared core hole of the anchor screw with minimal further drilling, was adhesively (Panavia F 21, Kuraray Europe, Hattersheim, Germany) inserted, core build‐up was performed (Figure 7E), and delivery of the final restoration took place (Figure 7F). A schematic overview of this procedure is shown in Figure 8.

The forced surgical extrusion is a relatively predictable and feasible procedure to save apparently unrestorable teeth, irrespective of patient age, which shows promising results with low complication rates [41, 43, 45]. One clinical study could demonstrate a success rate of 92.2% observation for the recalled teeth surgically extruded with the AES after a mean observation period of 3.1 years [41]. To avoid ankylosis, the atraumatic extraction method causes the least possible damage to the cementoblast layer on the root surface [23]. A brief extrusion time is advantageous, as it allows for the desired amount of extrusion to be achieved in a single session. This approach demonstrates low biological and prosthetic complication rates, good esthetics, and ready acceptance from the patient [43, 44]. In a clinical study, the surgical forced extrusion method with the benex system shows an overall biological success rate of 100% up to 4.8 years [43]. However, there is a risk of tooth fracture when screwing in the benex screw. For a proper comparison to the other extrusion techniques, clinical studies with longer follow‐up periods are also needed.

## 3 Discussion

Multiple techniques for tooth extrusion can be used as techniques to address the specific clinical challenge to restore severely compromised teeth with the aim to preserve those teeth. These methods are commonly regarded as a “conservative” alternative to more invasive procedures, like surgical crown lengthening or tooth extraction followed by implant placement or prosthetic tooth replacement. Other “conservative” therapy options, like surgical crown lengthening, are invasive as well and are often associated with a loss of attached gingiva and unpredictable esthetic outcomes due to alveolar bone resection [9]. The bone level of neighboring teeth is also often affected. However, it must be stated that clinical (long‐term) data on the survival and success of extruded teeth are currently scarce or not available, yet for most techniques [23].

Extrusion requires individualized planning based on defect level, apical status, attachment loss, and available vertical space to antagonists [2]. This is especially crucial for magnetic extrusion, for example, as the vertical height of the magnets, spacers, and the desired extrusion distance must be considered [8]. The possibility for a reliable fixation of the tooth during the retention phase should also be critically examined at the beginning. Additionally, to avoid unfavorable biomechanical forces, careful consideration of the crown‐to‐root ratio is essential during treatment planning [3, 21, 23]. The ratio does not shift as unfavorably compared to surgical crown lengthening, as extrusion only reduces the root length, while the crown height is not altered [10].

A further key advantage of extrusion can be the ability to preserve and even promote the individual migration of soft tissue. The orthodontic approaches in particular can result in hard and soft tissue gain in the coronal direction as tension is only applied to the surrounding tissues [17, 18, 19, 23, 30, 39]. The tension generated within the gingival fibers and periodontal ligament contributes to the coronal migration of both the soft and periodontal tissues. This type of force promotes marginal bone apposition through increased osteoblastic activity [23]. If this is not needed, a fibrotomy of the supracrestal periodontal fibers or root planing is necessary to facilitate tissue realignment and ensure successful healing in the new position [30].

The forced surgical extrusion provides immediate results by repositioning the tooth in a single appointment [23]. The technique involves the use of AESs to extrude the tooth and stabilize it in a more coronal position while preserving the cementum layer, a crucial factor for the success of the treatment. Multi‐rooted teeth with divergent roots and root anatomy are not compatible with atraumatic extraction [23]. However, the procedure reduces chairside time and offers good esthetic outcomes; there is a risk of tooth fracture due to the forces applied with the anchor screw. Although studies have reported ankylosis as a minor event, it is considered an adverse effect of surgical extrusion [45]. Nonprogressive root resorption in 30% of cases, followed by tooth loss or slight mobility, was reported [45]. Nevertheless, this approach is generally well accepted by patients, has a low failure rate, and minimizes the need for multiple appointments [44].

On the other hand, an extrusion with orthodontic appliances serves as an alternative treatment when contraindications exist for other extrusion techniques, for example in cases of vital teeth. Furthermore, this method seems to be most suitable in situations of vertical and horizontal alveolar defects characterized by gingival recession and/or papilla loss, which usually occur in association with periodontal disease or dental trauma [17]. The retention phase lasts between 4 weeks and 6 months, depending on the desired extrusion distance [17, 21].

The magnetic extrusion is a minimally invasive, cost‐effective, and simple, albeit time‐consuming treatment option with highly predictable results, utilizing controlled magnetic forces to reposition the tooth [12, 13, 14]. The force generated by the magnets decreases with the square of the distance between them [26]. This method offers advantages such as consistent force application and good control of force and eruption path at short distances, reducing the risk of damage to adjacent teeth and surrounding tissues [8, 14]. However, patients should be advised that discomfort and temporary tooth mobility may occur during the extrusion process. The control of direction, especially rotation around the tooth axis, can be controlled only to a certain extent. Despite its benefits, magnetic extrusion requires specialized equipment and wearing the equipment up to 24 h a day. So, this time‐consuming procedure needs a stringent compliance from the patients in particular. In cases of less vertical space and periodontal complications, the magnetic extrusion technique is not the option of choice [8]. As the force application of magnetic extrusion is comparable to that of orthodontic extrusion, similar success rates could be expected. Further studies with longer observation periods and a higher number of cases are needed.

In contrast, orthodontic extrusion with orthodontic appliances often involves full banding of the arches and application of occlusal forces on the extruded tooth, potentially causing intrusive forces on adjacent teeth. However, the continuous force transmission of the arches to the tooth, as well as an extrusion in a defined orthoaxial direction, is a clear advantage. This method is time‐intensive, typically requiring weeks to months, and is esthetically challenging. It also demands patient compliance, particularly with maintaining oral hygiene and attending weekly follow‐up visits performing supracrestal fibrotomy, if needed. A modified approach, known as FOE, stabilizes adjacent teeth with a rigid composite occlusal bar, simplifying the procedure for general practitioners [33, 38, 44]. This method works with significantly higher forces to be applied, in the presented case up to 10 N, compared to conventional orthodontic treatments or magnetic extrusion (≤ 1 N), reducing treatment time [14, 23, 27]. Crljenica et al. reviewed 10 cases of rapid orthodontic extrusion with conventional brackets and detected soft tissue migration following the crown movement for a total of 49%, while migration of hard tissues was only 18% of tooth movement [32]. Low risks of root resorption and ankylosis, such as the stability of the interproximal bone, are clear advantages of FOE [17, 26, 33]. In contrast to the magnetic extrusion, the vertical space is a less limiting factor and patients do not have to wear a splint during the extrusion period, which improves patient compliance. Moreover, there is no clinical evidence that magnets improve the outcomes compared to conventional orthodontic extrusion. Contraindications for all orthodontic extrusion include ankylosis, hypercementosis, root fractures, root resorption, short root length, and in multi‐rooted teeth, the risk of furcation exposure due to the treatment [20, 21, 24].

While tooth extrusion techniques in general offer conservative treatment alternatives, they are not without risks and complications. Regardless of the extrusion technique used, potential complications include ankylosis, root resorption, relapse, movement of adjacent teeth, and treatment failure followed by tooth extraction. Nevertheless, in the worst case, it must be stated that a treatment with tooth replacement by dental implants or other prosthetic solutions is still possible.

## 4 Conclusion

Various extrusion techniques offer clinicians the possibility of preserving severely damaged teeth and transferring them to a prosthetically safe prognosis while maintaining the biological width and considering the ferrule effect. Clinicians can select between various extrusion options based on patient‐, situation‐, and operator‐specific parameters. Medium‐term follow‐up data (up to 5 years) is not available for every technique, but existing studies show promising results. However, long‐term data on the survival and success of extruded teeth remain limited and urgently needed. Further clinical studies are essential to establish long‐term outcomes.

## Conflicts of Interest

The authors declare no conflicts of interest.

## Data Availability

The data that support the findings of this study are available from the corresponding author upon reasonable request.
